# Progress in Medicine

**Published:** 1897-03-27

**Authors:** 


### Progress in Medicine.

### FOODS.

The report on adulteration in London[1] shows some improvement, but the state of the milk trade is still very bad, for nearly one sample in five was condemned, and yet any that came out equal to the standard of poor genuine milk were accepted. Indeed, as the majority of all articles tested were purchased by inspectors in uniform the wonder is that so many frauds have been detected. Some alteration is needed both in the method of obtaining samples and in the standard adopted for milk. The water supply of London, too, suffered both in 1895 and 1896 from the long droughts, and in 1895 from the frosts, which brought to light serious defects. The greatly extended use of tomatos in late years, both in this country and in America, where their general employment only dates from the Civil War, has led to the discovery that to a few individuals they act as a violent poison. The tomato was originally brought from South America as a curiosity, and when some animals were found to devour the fruits, it gradaully gained acceptance as a relish and light food. A tomato disease has been described by W. T. English,[2] which he thinks attacks severely a quarter of the Americans who use this vegetable. The chief symptoms include irregular and rapid action of the heart, dyspnœa, endocarditis, as well as gastric irritation, pyrosis, severe nervous affections, and high tension of the pulse. Some few patients show symptoms of acute poisoning.

An apparent instance of tomato poisoning, which seems to be exceedingly rare in this country, was reported by F. Edgeworth.[3] The patient suffered from profound collapse, rapid pulse, &c., but finally recovered. The fruit was not, however, in good condition, and the symptoms may possibly have been due to ptomaines.

Somatose, according to R. Drews,[4] seems to have a specific action on the lacteal secretion. He tried it in the case of twenty women whose milk was deficient after other remedies had failed. In every patient a great increase of milk took place, much more than could be accounted for by the improvement in the patient's appetite and health. The dose administered was a teaspoonful given three or four times a day in milk.

**Alcohol.**—The treatment of inebriety requires a study of its cause in the particular patient, and of the injuries actually present and caused by it. Crothers[5] finds that immediate withdrawal is best, unless the patient fears it, and believes that he will sink under it. If carried out a sharp purge and hot bath, with massage and a twentieth of a grain of strychnia every four hours should be given. Hot beef-tea, milk, and coffee may be added. If the withdrawal is gradual, the strychnia may be given every two hours, and the bath and purge every day. Melancholia, tuberculosis, and neuritis are often the causes of the drink habit, and may spring up after its cure, as though concealed by its anæsthetic properties. Hence the treatment must include these affections if we would prevent a return of the craving for drink. There is, too, a danger in using opiates lest a drug craze be substituted for a drinking one.

The Commission upon the purity of beer[6] received evidence that over 55 million bushels of malt and 113,500 tons of sugar were used in 1895 for beer; that 42 pounds of malt and 28 of sugar were regarded as equal to a bushel of malt, for which 18 gallons of beer of a sp. gr. of 55 were expected. Various kinds of grain, saccharine, and glucose are used as substitutes, but the witnesses believed that no deleterious substances were employed at all.

A highly valuable paper on "Alcohol, its history, action, and uses," by Professor Binz, has been translated in the new Sydenham Society's series. It appears that distilled spirits date from the eighth century A.D., but they did not attract attention until the Renaissance, and came into use as a beverage later still. Binz considers that it increases the rapidity of the heart's action, partly through the natural compensation which follows upon dilatation of the vessels directly produced by it. But, besides this, alcohol increases the rate by its specific action on the heart. Another result of alcohol is to increase the quantity of air respired, but critics of Binz insist that this is an effort of nature to compensate for the decreased interchange of gases which goes on when alcohol is present in the blood.

Binz finds that it accelerates the motor activity of the stomach when given in small doses as well as the absorption of drugs like potassium iodide; though it is urged on the other hand that this quickened motor power is only due to the reduced excitability of the stomach and relaxation of the pylorus. Moreover, some observers point out that both peptic and pancreatic digestion are retarded by even moderate quantities. The old question as to the origin of fat in the body has been examined by Kaufman,[7] who lays down that the proteid elements of food supply part but not the whole of the fat by their metabolism. A large proportion of them is at once broken up and forms fat. Some of this is used at once as glycose to supply energy, some is stored as glycogen, while the rest of the product is deposited as fat. The proportions vary with the state and needs of the organism at the time. It will be recollected that Voit held that fat was exclusively derived from the proteid matter, and that hydro-carbon foods simply shielded the fat derived from the proteids against oxidation.

[1] Lancet, Nov. 7, 1896. [2] Dietetic and Hygienic Gazette, Nov. [3] Brit. Med. Journal, Feb. 27. [4] Centralblatt f. I. Med., 6 vii., 1896. [5] Med. Record, Oct. 24. [6] B. Med. Jour., Oct. 31. [7] Archives de Physiologie, Oct., 1896.

